# An audit of patients admitted to hospital in Nepal for COPD exacerbation

**DOI:** 10.1177/20503121221085087

**Published:** 2022-03-19

**Authors:** Marte Styrvold, Ane Jonette Heggset Sterten, Sudeep Shrestha, Subodh Dhakal, Ingunn Harstad

**Affiliations:** 1 Department of Public Health and Nursing, Faculty of Medicine and Health Sciences, Norwegian University of Science and Technology, Trondheim, Norway; 2 Department of Internal Medicine, Dhulikhel Hospital, Dhulikhel, Nepal; 3 Department of Internal Medicine, Kathmandu Medical College, Kathmandu, Nepal; 4 Department of Pulmonary Medicine, St. Olav’s University Hospital, Trondheim, Norway

**Keywords:** COPD, acute exacerbations of COPD, COPD-exacerbations, GOLD guidelines, low-income countries, Nepal

## Abstract

**Objectives:**

Chronic obstructive pulmonary disease is a large and increasing problem in low- and middle-income countries; Nepal is no exception. We aimed to obtain information on patient characteristics and the level of care provided to patients admitted for acute exacerbation of chronic obstructive pulmonary disease in two Nepalese hospitals and to compare the given care with the Global Initiative for Chronic Obstructive Lung Disease guidelines.

**Methods:**

This was a cross-sectional, observational, descriptive study. All patients admitted to two Nepalese hospitals due to acute exacerbation of chronic obstructive pulmonary disease between 18 February and 5 April 2019 were asked to participate.

**Results:**

In total, 108 patients with a median age of 70 years participated. Fifty-three (42.7%) were male, 80 (74.8%) were former smokers, and 46 (45.1%) were farmers. Using the Global Initiative for Chronic Obstructive Lung Disease A-D classification, 97 (90.6%) of the patients were classified in group D. All the patients received supplementary oxygen treatment and 103 (95.4%) were treated with short-acting beta2 agonists. A total of 105 (97.2%) patients received antibiotics, and 80 (74.5%) received systemic corticosteroids. The majority was discharged with triple therapy including long-acting muscarinic antagonist, long-acting beta2 agonist, and inhaled corticosteroids, and 72 (75.8%) were discharged with long-term oxygen treatment.

**Conclusion:**

All elements of the Global Initiative for Chronic Obstructive Lung Disease guidelines were applied. However, due to a lack of information, it cannot be concluded whether the treatment was provided on the correct indications. The average patient received almost all the treatment alternatives available. This might indicate a very sick population or over-treatment.

## Introduction

Chronic obstructive pulmonary disease (COPD) is a substantial health issue and a leading cause of mortality and morbidity globally. In 2015, 3.2 million people died from COPD worldwide. The main risk factor worldwide for developing COPD is exposure to tobacco smoke.^1^

A meta-analysis indicated that exposure to indoor air pollution due to biomass smoke is also strongly associated with COPD.^2^ In Nepal, 98% of the households in rural areas still rely on open fires using biomass fuel, without proper ventilation.^3^ According to the World Bank, Nepal is a low-income country.^4^ Although more than 90% of COPD deaths occur in low- and middle-income countries, the majority of information on COPD prevalence, mortality, and morbidity comes from high-income countries.^5^ In an analysis from 2015, Nepal was ranked among the four countries with the highest age-standardized disability-adjusted life year rates due to COPD.^1^

The Global Initiative for Chronic Obstructive Lung Disease (GOLD) develops and renews management guidelines for COPD. Acute exacerbation of chronic obstructive pulmonary disease (AECOPD) is defined by GOLD as ‘an acute worsening of respiratory symptoms that results in additional therapy’.^6^ The diagnosis is based on clinical findings such as increased dyspnea, cough, and/or sputum production. AECOPD is an important event in the course of the disease and has a negative impact on the patient’s quality of life and the prognosis of the disease.^6^ In addition, a high economic burden is associated with the event.^7^ Differences between hospitals in terms of care strategies and results obtained have already been revealed in several studies, for example, from the United Kingdom and Spain.^8–10^

Suboptimal in-hospital therapy for AECOPD is expected to lead to more frequent exacerbations, recurrent hospitalization, and, consequently, increased risk of morbidity and mortality.^11^ For this reason, it is important to obtain information regarding in-hospital management to address how to improve the treatment of patients with AECOPD. Quality improvement interventions could make a significant difference to patient care. A lack of earlier research in low-income countries accentuates the importance of performing the research, as it may reveal potential improvements and raise further research questions. Thus, we aimed to obtain information on patient characteristics and the level of care provided to patients admitted for AECOPD in two Nepalese hospitals. By comparing the current practice with the recommendations of the GOLD guidelines, areas that may be improved can be targeted.

## Methods

The audit was designed as a cross-sectional, descriptive, observational study.

### Inclusion criteria

All patients requiring hospital admission due to AECOPD between 18 February and 5 April 2019 at two Nepalese hospitals were asked to participate. In rural areas of Nepal, COPD diagnosis is made clinically. Patients with a prior clinical-based COPD diagnosis were therefore included.

### Study site

The study was performed at Dhulikhel Hospital (DH) and Kathmandu Medical College and Teaching Hospital (KMC). DH is an independent, non-profit institution which covers a population of approximately 1.6 million people located in a small town about 1-h drive from Kathmandu University School of Medical Sciences.^12^ DH has 475 beds included 55 beds in the medical ward where pulmonary patients are admitted. After discharge from AECOPD, all patients are offered a follow-up in the out-patient department where spirometry is available. KMC is a private medical college located in Kathmandu with 675 beds. In the medical ward up to two-thirds of the 54 beds are occupied by pulmonary patients. Both institutions are affiliated with Kathmandu University.^13^

### Data collection

The data collection sheet in Supplemental Figure 1 was used to collect the data. The sheet contained five different parts: treatment during hospital stay, examination during hospital stay, comorbidity, discharge information, and a questionnaire. The questionnaire contained questions regarding socioeconomic status, risk factors, previous history, and the Modified Medical Research Council (mMRC) dyspnoea scale.^14^ Age and sex were also registered. A pilot study was conducted at DH a week before the data collection started. To ensure comprehension, the questionnaire was read to the patient in Nepalese by intern doctors. Information regarding treatment, examinations, and outcome was collected from medical records. The patients were also asked to give permission for a follow-up call to themselves and/or their next of kin 6–7 months after discharge. From this call, the mortality at 3 months and at the time of the call was assessed.

### Analyses

Differences in dichotomous variables were evaluated using Pearson’s chi-square test (χ^2^) or Fisher’s exact test. Normally distributed parameters were analysed using Student’s *t*-test (independent *t*-test). Non-normally distributed parameters were evaluated using the nonparametric Mann–Whitney *U* test, Kruskal–Wallis test, or Spearman’s rank correlation coefficient. SPSS, version 25, for Mac (IBM Corp., Armonk, NY) was used. All tests were two-tailed; a *p*-value of < 0.05 was considered significant. The results are expressed as count (n), proportion (%), and median and interquartile range.

GOLD guidelines^6^ were used to classify patients and comparing COPD interventions. Even though the patients in our study had a clinically based diagnosis as they were diagnosed without spirometry, patients were classified into GOLD class A-D by the variables symptom score and moderate or severe exacerbation history and assessed on the variables in Table 2.

### Ethics

The patients were asked to provide informed consent before inclusion in the study. An information sheet was read to the patients in Nepalese and oral consent was given. Participants who were able to write, signed the sheet. The participants could withdraw from the study at any time. Ethical approval was obtained from the Regional Committees for Medical and Health Research Ethics in Norway and the Institutional Review Committee at both DH and KMC in Nepal prior to the study.

## Results

### Demographics/characteristics

**Figure 1.**
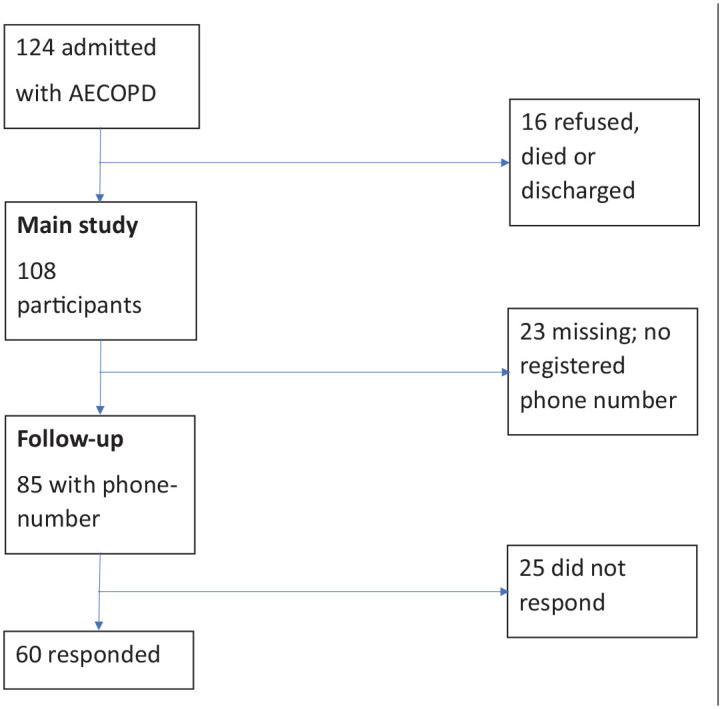
Flow sheet of study participants.

A total of 124 patients were registered (Figure 1). The median age was 70 years, and 53 (42.7%) were men. Sixteen patients were not included in the remainder of the analysis as they refused to participate, had died, or were discharged before they could participate. Of the remaining 108 patients, the median length of hospital stay was 5 days. Of all the patients, 17 (15.7%) were current smokers, and 80 (74.8%) were former smokers. In addition, 99 (92.5%) of the study participants had been exposed to biomass fuel smoke, and most of them had been exposed since childhood. Altogether 46 (45.1%) participants were farmers. Thirty patients (28%) had hypertension, which was the most frequent comorbidity. A median of one comorbidity was estimated. Based on the assumption that those with no schooling were illiterate, 73% of the study population were illiterate (Table 1).

**Table 1.** Patient characteristics.

Category	Variable	Patients, *n*	%	Median	IQR
Sociodemographic characteristics	Total, n	124	100		
	Age (years)			70	18
	Sex (females)	71	57.3		
	Privileged ethnic group	85/95	89.5		
	BMI (kg/m^2^)	20	6	20	6
	Urban living	35/108	32.4		
	Education	12/107	11.2		
	Illiterate	73/104	70.2		
Work	Farmer	46/102	45.1		
	Retired/old	46/102	45.1		
	Other	10/102	9.8		
Smoking status	Never-smoker	10/107	9.3		
	Current-smoker	17/107	15.9		
	Pack years			33.3	46.9
	Ex-smoker	80/107	74.8		
	Pack years			19	34
Biomass fuel smoke	Exposed	99/107	92.5		
	Years			50	20
Hospitalization AECOPD	Earlier admitted	74/108	68.5		
	Number in total			3	6
	Number last year			1	1
Category mMRC	0–2	36/107	33.7		
	3–4	71/107	66.3		
Gold A-D	C	10/107	9.4		
	D	97/107	90.6		
Count of comorbidity DAMA				1	1
		9/103	8.7		
Comorbidity	None	52/107	48.6		
	CHD	4/107	3.7		
	Hypertension	30/107	28.0		
	Diabetes	22/107	20.6		
	Heart failure	3/107	2.8		
	Others	15/107	14.0		

IQR: interquartile range; BMI: body mass index; AECOPD: acute exacerbation of chronic obstructive pulmonary disease; mMRC: Modified Medical Research Council; DAMA: discharge against medical advice; CHD: coronary heart disease; DH: Dhulikhel Hospital; KMC: Kathmandu Medical College.

In all, 74 (68.5%) had been admitted previously due to AECOPD, and they had a median of one admission the last year.

### Adherence to GOLD guidelines

The patients were classified according to the GOLD A-D classification; due to their present hospital admission, all the patients were either categorized into group C or D and due to an mMRC score of ⩾2, 97 (90.6%) of the patients ended up in group D.

Only 11 (10.8%) said they had performed spirometry the last year, and only one (0.9%) had the results available when admitted. A total of 94 (87.9%) had at least one recorded arterial blood gas (ABG) result. In all, 105 (99.1%) of the patients had undergone chest X-ray. Sputum culture was performed in 77 (76.5%) of the cases. However, 93 (89.4%) had undergone a sputum test including microscopy of a gram stain and an acid-fast bacilli smear.

All the patients received oxygen, with a median of 3 L during the hospital stay. On the day of arrival, 46.4% of those receiving supplementary oxygen treatment had a saturation above 92%. Overall, 103 (95.4%) were treated with short-acting beta2 agonists (SABAs), and 104 (96.3%) with either short-acting muscarinic antagonists or SABAs. Among those treated with short-acting bronchodilators (SABDs), 103 (99.0%) received the treatment through a nebulizer. Systemic corticosteroids, either by injection or orally, were administered to 80 (74.8%) of the participants. Antibiotics were given to 105 (97.2%) of the patients, and the initial antibiotic treatment consisted most frequently of a macrolide and a third-generation cephalosporin. In all, 33 (31.7%) received non-invasive ventilation treatment through bilevel positive airway pressure (BiPAP), while 4 (3.8%) received invasive mechanical ventilation (IMV) treatment.

In total, 93 (96.9%) were discharged with long-acting bronchodilators (LABDs), of whom 85 (91.4%) were discharged with both long-acting muscarinic antagonists (LAMAs) and long-acting beta2 agonists (LABAs). In all, 92 (95.8%) were discharged with inhaled corticosteroids (ICS). The number of patients discharged with methylxanthines was 37 (38.9%) (Table 2).

**Table 2.** Summary of adherence to AECOPD GOLD guidelines.

GOLD guidelines	In total		DH		KMC		*p*
	%	*n*	%	*n*	%	*n*	
Under hospitalization							
Spirometry results^a^	0.9	1/101	0	0/56	2.2	1/45	0.262
Arterial blood gas	87.9	94/107	82.5	47/57	94.0	47/50	0.068
Chest X-ray	99.1	105/106	98.3	56/57	100	48/48	0.361
Sputum culture	75.5	77/102	80.4	45/56	69.6	32/46	0.559
Oxygen therapy	97.9	93/95	96.3	52/54	100	41/41	0.504
SABD	96.3	104/108	93.1	54/58	100	50/50	0.058
Systemic corticosteroids	74.8	80/107	74.1	43/58	75.5	37/49	0.871
Antibiotics	97.2	105/108	96.6	56/58	98.0	49/50	0.648
NIV	31.7	33/104	34.5	19/55	28.6	14/49	0.541
IMV^b^	3.8	4/104	0	0/55	8.2	4/49	0.031
Prescribed at discharge							
LABD	96.9	93/96	100	52/52	93.2	41/44	0.093
SABD^b^	30.2	29/96	53.8	28/52	2.3	1/44	<0.001
LTOT^b^	75.8	72/95	67.3	35/52	86.0	37/43	0.034
Methylxanthine (doxophylline)	38.9	37/95	45.1	23/51	31.8	14/44	0.186

AECOPD: acute exacerbation of chronic obstructive pulmonary disease; DH: Dhulikhel Hospital; KMC: Kathmandu Medical College; GOLD: Global Initiative for Chronic Obstructive Lung Disease; SABD: short-acting bronchodilators; NIV: non-invasive ventilation; IMV: invasive mechanical ventilation; LABD: long-acting bronchodilator; LTOT: long-term oxygen therapy; SABD: short-acting bronchodilator.

a Spirometry results available at admission.

b Significant.

### Comparison between hospitals

The distribution between KMC and DH was 57 (46.0%) and 67 (54.0%) patients, respectively. At KMC, 43 (86.0%) arrived from rural areas compared to 30 (51.7%) at DH. A larger percentage of patients were classified in mMRC groups 3 and 4 at DH compared to KMC (*p* = 0.024). At DH, 56 (96.6%) of the patients were categorized into GOLD group D, while at KMC, 41 (83.7%) were in this group (*p* = 0.023). At KMC, ABG had been measured in 47 (94.0%) of the cases, and at DH, in 47 (82.5%) of the cases (*p* = 0.034). At DH, 19.5% had oxygen saturation ⩾80% without oxygen treatment, compared to 47.6% at KMC (*p* = 0.009). Overall, 72 (75.8%) of the patients were discharged with long-term oxygen treatment (LTOT). At DH, 35 (67.3%) of the patients received LTOT compared to 37 (86.0%) at KMC (*p* = 0.034). In all, 29 (30.2%) of the patients were discharged with SABD. There was also a significant difference between the hospitals, where SABD was received by 28 (53.8%) at DH compared to one (2.3%) at KMC (*p* < 0.001). There was a significant difference between the hospitals regarding the length of hospital stay even after excluding IMV patients. At KMC, the median length of stay was 7 days compared to 4 days at DH (*p* < 0.001).

### Mortality

Contact information for phone calls was collected from 85 participants among whom 60 participants or their relatives responded. Six to seven months after discharge when the phone call was done, altogether 22 (36.7%) had died, and 12 (21.1%) had died within 3 months of discharge. Age (*p* = 0.002) and a high grade of mMRC (*p* = 0.027) were variables associated with mortality.

## Discussion

### Main findings

This is the first internationally published audit on in-hospital care for AECOPD in Nepal. The management of AECOPD was compared with the international GOLD guidelines and between the two participating hospitals. The study found generally good adherence to the GOLD guidelines, but also revealed probable overuse of some treatment alternatives. Nearly all participants had antibiotic treatment during their stay and a great number was discharged with LTOT. Methylxantines were prescribed on a regular basis even though they are not recommended in the GOLD guideline. Thus, there are room for improvements, but also a need for further research to uncover more information about the treatment and the patient group. To discuss this audit further in relation to the GOLD guidelines and previous studies, we will look into in-hospital treatment, care on discharge, comparing the two hospitals, and finally mortality.

### In-hospital treatment

ABG reflects the severity of the exacerbation and is a strong predictor of in-hospital mortality.^6,11,15^ Eighty-eight percent of the participants had at least one ABG result recorded during hospitalization. In a European study, including 13 countries and 16,018 patients, 82.5% had an ABG measurement recorded.^16^ The result is at European level but considering how seriously ill these patients were and that 98% of the patients received oxygen, there were probably indication for ABG for all the patients. Most of the participants received oxygen therapy with a median volume of 3 L. GOLD recommends a target saturation of 88%–92% in AECOPD.^6^ On the day of arrival, 46.4% with supplementary oxygen had a saturation >92%. This may indicate oxygen over-treatment.

All except one patient had undergone chest X-ray. The chest X-ray consisted of a frontal view only, and without a written report, this may likely lead to less reliable interpretations.

Only 80 (74.8%) of the participants received systemic steroids during the hospital stay. Systemic steroids for treating patients with AECOPD have been demonstrated to reduce the rate of treatment failure and breathlessness.^17^ It is questionable that almost everyone received oxygen therapy upon admission and LTOT at discharge; however, only three-quarters received systemic steroids during the hospital stay.

There was good adherence to the guideline considering receiving SABA during hospitalization. GOLD emphasizes that SABD should be administered initially; however, the study did not investigate when the treatment was initiated.

Antibiotics were administered during in-hospital care more frequently compared to what is given in Scandinavia.^18^ Until recently, bacteria were believed to be responsible for the majority of exacerbations. Following the implementation of polymerase chain reaction, it is now estimated that viruses are associated with half of all exacerbations.^19,20^ Considering the unavailability of viral throat swabs and urinary legionella and strep pneumoniae antigens, the criteria for antibiotic therapy were not registered, suggesting that a proportion of ideal candidates for antibiotic therapy could not be measured. Nevertheless, the overuse of antibiotics is possible.

### Care on discharge

Almost all the patients were discharged with minimum maintenance medication based on GOLD recommendations. In both groups C and D, triple therapy with LAMA, LABA, and ICS represented the most frequently prescribed therapy. GOLD recommends LAMA as the only initial maintenance medication in group C. The observed high frequency of triple therapy might be due to a lack of efficacy of the initial treatment or a matter of standard procedure.

Less than one-third of the participants were discharged with SABD. The degree in which the hospitals prescribed rescue SABD was significantly different. This disparity could be due to different attitudes towards prescribing rescue treatment. At KMC, one (2.3%) received SABD at discharge. One of the explanations for this low number was that they believed the patients already had SABD at home.

A large percentage of the patients were discharged with LTOT. The proportion of patients who received LTOT at DH was significantly lower than at KMC. The data points to DH as the hospital having patients with the poorest saturation; however, comorbidities were not significantly different. The variability could be due to differences in adherence to the guidelines and attitudes towards prescribing LTOT treatment, economic reasons, or availability. The British Thoracic Society^21^ guideline for home oxygen recommends that patients should undergo formal assessment for LTOT after a period of stability of at least 8 weeks from their last exacerbation. This implicates a massive overuse of LTOT at discharge. It is also unclear if the patients actually use the LTOT as prescribed.

The use of xanthine derivatives is controversial as it has modest effects, a small therapeutic ratio, and a risk of ventricular arrhythmias.^6^ In the GOLD guidelines they are not part of the main treatment pathway and not recommended during exacerbations. However, they are mentioned to have a small bronchodilator effect and modest symptom benefits. Doxophylline (a xanthine derivative) was prescribed to 37 (38.9%) of the patients at discharge. Since almost all were discharged with LABD, it seems unnecessary to add doxophylline. A possible explanation for the high prescription rate is that doxophylline is an inexpensive tablet which is easy to use.^22,23^

### Comparison between hospitals

A significant difference in the length of stay between the hospitals was revealed. DH had a higher number of patients in GOLD group D in addition to a larger proportion of patients with saturation below 80% which suggests that a high symptom burden might not be the reason for the difference. Patients undergoing IMV stayed longer at the hospital. At KMC, four patients underwent IMV compared to none at DH. Even when accounting for IMV, the difference in the length of hospital stay remained significant, suggesting that it is not the only reason. At KMC, a significantly higher proportion of participants lived in rural areas which could have made it necessary to extend the hospital stay to ensure that the patients were stable when discharged. Variables not investigated, such as financial reasons and patient load, could also have contributed to the difference.

### Mortality

Compared to studies from high-income countries, the mortality after 3 months of follow-up was high (21.1% in our study). Studies from high-income countries have shown a mortality rate ranging from 12.4% to 15.3% after 90 days of follow-up.^8,24^ A cohort study from a hospital in Nepal including 22,000 patients investigated the 90-day mortality after entering the emergency department. The highest mortality was seen in individuals with known chronic lung disease among whom 32% died within 90 days.^25^ This suggests that the finding in our study regarding mortality is likely to be underestimated.

The majority of hospitalized individuals were illiterate, which may cause poor compliance, self-care, and follow-up attendance. Cultural attitudes towards traditional western medicine and poor personal economy can make the threshold for seeking hospital care higher. Together, these factors can result in a population with poorer health compared to high-income countries and have an impact on the mortality and provided treatment.

### The strengths and limitations

This study has several limitations. First, the sample size was small, making the study less valid and reliable and lowering the ability to detect possible effects and/or deviations. The diagnosis of COPD is based on spirometry results. However, spirometry results were available for only one patient on admission; therefore, patients were recruited based on a clinical diagnosis of COPD. Since other conditions could have similar symptoms, this may have led to both over- and underdiagnosis in addition to a selection bias. Another limitation is that the questionnaire was not validated before this study, except through a small pilot. This could lead to inaccurate questions that did not give a reply on what we wanted to know. On some occasions, when questioning the patient, the family responded on behalf of the patient, which also introduced some uncertainty regarding the validity of the responses.

The study, nevertheless, also has strengths. The study was planned in conjunction with and guided by physicians working at each hospital who have knowledge of the hospital system and the patient group. Data were collected from two hospitals, making the population more robust concerning confounding and bias. Almost all the patients wanted to participate in the study. The same person at each hospital, with a few exceptions, questioned the patients and collected data together with the authors. Thus, variations based on the interpretation of the questionnaire and administration to the patient were limited.

## Conclusion

All elements of the GOLD guidelines were used in both hospitals. The audit also revealed that the average patient received almost all the available treatment alternatives. This might indicate that the population is very ill or suggest a therapy strategy which is insufficiently adapted to the individual, making over-treatment likely. It is necessary to assess if a patient needs and would benefit from all the treatment alternatives available, especially concerning the cost and health effects. We will in particular question the use of methylxanthines and the indication for oxygen therapy on discharge. However, due to a lack of information, it cannot be concluded whether the treatment was administered on the correct indications. Further extended studies are required to reveal whether treatment is provided according to the guidelines and whether our findings are representative for other hospitals in Nepal.

## Supplemental Material

sj-docx-1-smo-10.1177_20503121221085087 – Supplemental material for An audit of patients admitted to hospital in Nepal for COPD exacerbation Click here for additional data file. Supplemental material, sj-docx-1-smo-10.1177_20503121221085087 for An audit of patients admitted to hospital in Nepal for COPD exacerbation by Marte Styrvold, Ane Jonette Heggset Sterten, Sudeep Shrestha, Subodh Dhakal and Ingunn Harstad in SAGE Open Medicine
