# Supplementary material for: An audit of patients admitted to hospital in Nepal for COPD exacerbation
Source: SAGE Open Med. 2022 Mar 19;10:20503121221085087. doi: 10.1177/20503121221085087 (PMC8935543; doi:10.1177/20503121221085087)
Supplement: sj-docx-1-smo-10.1177_20503121221085087 – Supplemental material for An audit of patients admitted to hospital in Nepal for COPD exacerbation [file sj-docx-1-smo-10.1177_20503121221085087.docx]

| **COPD EXACERBATION IN NEPAL** | | | | | **LINK-KEY:** |
| --- | --- | --- | --- | --- | --- |
| **ENGLISH** | | | **NEPALI** | | |
| Hospital: | | Dulikhel KMC |  | | |
| Gender: | | Female Male |  |  |  |
| Age: | |  |  |  |  |
| Participate in this study | | Yes No |  |  |  |
| **QUESTIONNAIRE:** | | |  | | |
| 1. Which ethnic group do you belong to?  Brahmin and Chetri Janajati Dalit Newars Others | | | जनसांख्यिकी तथ्यांक: १.तपाई कुन समुदायमा बस्नुहुन्छ ?  ब्रह्मिन, क्षेत्री, जनजाती, दलित, नेवार, अन्य | | |
| 2. Have you ever been to school? Yes No  2.1 If yes, how many years have you been to school? ______  2.2 If no, can you read and write? Yes No Some | | | २.तपाई कहिले स्कूल जानुभाको छ ? Yes No  २.१यदि जानुभाको छ भनि :कति वर्ष जानु भयो?______  २.२.यदि छैन: तपाई पडन लेख्न सक्नुहुन्छ? Yes No Some | | |
| 3. How are you earning your living? | | | ३.तपाई जिबिकोपार्जनको लागि के गर्नुहुन्छ?  (ज्यालादारीकाम ,ब्यापार , खेतिकिसानी , बृध , अन्य ) | | |
| **Risk factors**  4. Are you a current smoker? Yes No  4.1 If yes, how many cigarettes per day? _______  and for how many years? _______    4.2 If no, have you been smoking earlier in your life? Yes No  If yes, how many cigarettes per day? _______  and for how many years? _______ | | | **जोखिम कारकहरू:**  ४.हालसाल चुरोट सेबन गर्नुहुन्छ? Y es No  ४.१येदि गर्नुहुन्छ भनि :दिनमा कति ओटा गर्नुहुन्छ? _____  कति वर्षके गर्नलाको? _____  ४.२येदि गर्नु हुन्न भनि :पहिले कहिले गर्नु भाको छ? Yes No  येदि गर्नुभाको छ भनि दिनमा कति सेवन गर्नु हुन्थ्यो? ___  र कति वर्ष गर्नुभयो? _____ | | |
| 5. Have you ever been exposed for biomass fuel? Yes No  5.1. If yes: for how many years? _______ | | | ५.कहिले दाउरा गुइठा पर्योग गर्नु भाकोछ? Yes No  ५.१यदि गर्नु भाको छ भनि कति वर्ष गर्नु के? _______ | | |
| 6. What kind of work have you done in your life? ____________  And of how many years? ________    6.1 Possible exposure to:  gasses stone dust other dust other air pollution | | | ६.तपाईले जिबनमा कुन कुनपेशा गर्नु भयो र कति वर्ष गर्नु भयो? _____________  ६.१कहिले ढुंगा फोहोर हावा र धुलो धुवामा गर्नी कम गर्नुभाको छ? | | |
| **Previous history**  7. Have you been admitted to any hospital for COPD before?  Yes No 7.1 If yes, how many times altogether? _______  how many times last year? _______ | | | **अघिल्लोइतिहास**  ७.पहिला कहिले कुनै अस्पतालमा दमको कारणले भर्ना हुनुभाको छ? Yes No ७.१यदि छ भनि कति पटक? ______  गयको वर्ष कति पटक भर्ना हुनु भयो? ______ | | |
| 8. Have you ever been treated for tuberculosis? Yes No  8.1. if yes, was that before or after you got your diagnosis of  COPD?  Before After | | | ८.तपाईलाही कहिले क्षय रोग लागेको छ र उपचार गर्नु भाको छ? Yes No  ८.१येदि छ भनि दम हुनु पहिला वदमवाई सके पछि?  Before After | | |
| **MMRC – Grading of breathing problems** | | | **MMRC - Grading of breathing problems** | | |
| Grad 0 | Dyspnea only with stenuous exercise | |  | गारो कम गर्दा मात्र सास फेर्न गारो हुन्छ? | |
| Grad 1 | Dyspnea when hurrying or walking up a slight hill | |  | छिटो हिड्दा वाह उकालोमा गारो हुन्छ? | |
| Grad 2 | Walks slower than people of the same age because of dyspnea or has to stop for breath when walking at own pace | |  | तपाईको संगी बराबर हिड्न सक्नुहुन्न वाह हिड्दा हिड्दै रोकिनुपर्छ? | |
| Grad 3 | Stops for breath after walking 100 yards(91m) or after a few minutes | |  | सय मीटर हिडेपछि सास फेर्न गारोभएर रोकिनु पर्छ कि पर्दैन? | |
| Grad 4 | Too dyspneic to leave house or breathless when dressing | |  | सास फेर्न गारो भएर घरबाट निस्कन नसक्नी लुगा फेर्न नसक्नी? | |

| **COPD EXACERBATION IN NEPAL** | **LINK-KEY:** |
| --- | --- |
| **TREATMENT DURING HOSPITAL STAY**  Oxygen support in liters: __________  Ventilatory support Ventilator NIV (non-invasiv ventilation) CPAP (continuous positive airway pressure)  Inhalation treatment SABA (short acting beta agonist) Anticholinergica Others  Antibiotics: No Yes, witch type: _____________________  Steroids: No Yes, witch type: _____________________ | |
| **EXAMINATION DURING HOSPITAL STAY**  Chest X-ray: No Yes. If yes: Normal Pathology: ______________________  Blood test: Leukocytes Differential count CRP  Sputum test: Tuberculosis Other infections  Cultur: Tuberculosis Other infections | |
| **KNOWN COMORBIDITIES**  Coronary heart disease Hypertension Diabetes mellitus Hearth failure Other | |
| **ADDED EXAMINATION FOR STUDY PURPOSE**  Oxygen saturation   - On admission/added in the study: ______ Without O2? Yes No - At discharge : _______ Without O2? Yes No   Spirometry  Result from spirometry done at discharge Results from spirometry done the last year   - Weight :______Kg - Height :______m - FVC :______ - FEV1 :______ - FEV1/FVC : ______L ______% | |
| **TREATMENT ON DISCHARGE**  Oxygen support in liters: __________  Ventilatory support Ventilator NIV(non-invasiv ventilation) CPAP (continuous positive airway pressure)  Inhalation treatment SABA (short acting beta agonist) Anticholinergica Others  Other inhalation Steroids LAMA (long acting muscarinic antagonist) LABA (long acting beta agonist)  Antibiotics: No Yes, witch type: __________  Steroids: No Yes, witch type: __________  Other respiratory related medicine: _______________ | |
| **ON DISCHARGE**  Number of days stayed in hospital: _____________  Diagnosis on discharge : _________________________________________________________  Discharge after doctor’s decision: Yes No.  If no, discharge after family’s decision (against doctors advise): Yes No  Status : Better Worse Same Died | |
